# T-Shaped Microfluidic Junction Processing of Porous Alginate-Based Films and Their Characteristics

**DOI:** 10.3390/polym11091386

**Published:** 2019-08-23

**Authors:** Betul Mutlu, Muhammad Farhan, Israfil Kucuk

**Affiliations:** 1 Graduate School of Natural and Applied Sciences, Bursa Technical University, Bursa 16310, Turkey; 2 Department of Pharmaceutics, Bahauddin Zakariya University, Multan 60800, Pakistan; 3 Institute of Nanotechnology, Gebze Technical University, Gebze 41400, Turkey

**Keywords:** porous films, T-shaped microfluidic junction, coatings, bioengineering applications, thin film

## Abstract

In this work, highly monodisperse porous alginate films from bubble bursting were formed on a glass substrate at ambient temperature, by a T-shaped microfluidic junction device method using polyethylene glycol (PEG) stearate and phospholipid as precursors in some cases. Various polymer solution concentrations and feeding liquid flow rates were applied for the generation of monodisperse microbubbles, followed by the conversion of the bubbles to porous film structures on glass substrates. In order to compare the physical properties of polymeric solutions, the effects of alginate, PEG stearate (surfactant), and phospholipid concentrations on the flowability of the liquid in a T-shaped microfluidic junction device were studied. To tailor microbubble diameter and size distribution, a method for controlling the thinning process of the bubbles’ shell was also explored. In order to control pore size, shape, and surface as well as internal structure morphologies in the scalable forming of alginate polymeric films, the effect of the feeding liquid’s flow rate and concentrations of PEG-stearate and phospholipid was also studied. Digital microscopy images revealed that the as-formed alginate films at the flow rate of 100 µL·min^−1^ and the N_2_ gas pressure of 0.8 bar have highly monodisperse microbubbles with a polydispersity index (PDI) of approximately 6.5%. SEM captures also revealed that the as-formed alginate films with high PDI value have similar monodisperse porous surface and internal structure morphologies, with the exception that the as-formed alginate films with the help of phospholipids were mainly formed under our experimental environment. From the Fourier-transform infrared spectroscopy (FTIR), Differential scanning calorimetry (DSC) and X-ray diffraction (XRD) measurements, we concluded that no chemical composition changes, thermal influence, and crystal structural modifications were observed due to the T-shaped microfluidic junction device technique. The method used in this work could expand and enhance the use of alginate porous films in a wide range of bioengineering applications, especially in tissue engineering and drug delivery, such as studying release behaviors to different internal and surface morphologies.

## 1. Introduction

There are various strategies for the production of highly ordered biodegradable and biocompatible porous film structures for tissue engineering and drug delivery applications. Therefore, it is of interest to produce highly monodisperse porous films having the desired porosity that brings about a suitable platform to immobilize biomolecules and cells, and to load drugs in different morphologies [1,2]. Polymeric scaffolds and film structures like alginate-based porous films have many unique advantages for tissue engineering and drug delivery systems, like biodegradable film drug carrier treatments [3]. For instance, a couple of significant advantages could include the controlled release to its target field at the suitable period of time and the desired amount of drug loading ability to provide a larger surface area of application [4]. Additionally, in most porous film structures, control of the porosity of the polymeric films plays an important role in either allowing cells to be seeded within the film surfaces in larger pores or inhibiting cell infiltration in smaller pores [5]. A substantial amount of film porosity is often necessary to allow for cellular activities and interactions, including cell signaling [6]. Porous film structures with a larger pore size distribution and various pore geometries would therefore benefit from a highly ordered architecture that enhances its mechanical properties and resistance to compressive stresses compared to porous films with a low polydispersity index (PDI) [7].

Various traditional technologies that produce porous structures in biomedical applications, including electrospraying [8,9], freeze drying, and particle leaching, have demonstrated success in film formation; however, a wide pore size distribution was obtained which is not suitable for biomedical applications [4]. An alternative emerging technology, bioprinting, has demonstrated the ability to control pore size and distribution and the microarchitectural features in biofilms, forming engineered tissues with structure and function similar to native tissues; however, this can only be achieved in microscale and the methods are time consuming [10,11]. Thus, we believed that microfluidic techniques including a T-shaped microfluidic junction device, when compared to the others, could overcome the previous application limitations, such as lack of pore size, shape, and surface control in the scalable formation of polymeric films [7,12,13,14,15]. For example, Elsayed et al. almost obtained good control over the pore structure of the resultant films by embedding designed nanoparticles using a T-junction technique [15]. This could expand their bioengineering applications, in particular when drug delivery necessary for eye disease treatments is desired. Furthermore, this could provide a chance for the use of a wide category of polymers (i.e., naturally derived polymers) that lose their internal structure as a result of harsh processing [4].

Naturally derived polymers such as hyaluronic acid, chitosan, and alginate are characterized by highly organized chemical structures [16] that can add additional desirable features to their therapeutic use, making them widely used in biomedical applications [17]. Attention has been paid to the use of such naturally derived materials in wound dressings and enzyme, protein, or drug delivery, as well as film structures for tissue engineering or disease treatments [3]. There is therefore a need to understand how to formulate effective porous film structures from biocompatible and cost-friendly naturally derived polymers and surfactants [2]. Sodium alginate (SA) is a biodegradable polymer derived from marine brown algae; its simplest fundamental unit is related to cellulose and the structural configuration is best represented by (1,4)-β-D mannuronate and (1,3)-α-l-guluronate residues [18]. It has been widely used in biomedical applications due to its characteristics such as the carboxylic groups contained in every repeating unit of its structure that has α and β configurations which improve its biocompatibility [1]. Moreover, alginate holds great interest as a potential biopolymer film or coating component due to its unique colloidal properties, which include thickening, stabilizing, emulsion stabilizing, suspending, gel producing, and film forming [19]. Polyethylene glycol (PEG) stearate, as a surfactant material, is a widely used hydrophilic polymer, because of its bonding structure which indicates the influential effect of the hydroxyl end groups on the chemical and physical properties of these molecules [20]. Moreover, PEG stearate is one of the suitable surfactant materials for use in various applications in tissue engineering and drug delivery systems [7]. In order to produce porous film structures, phospholipids are also widely used hydrophilic polymer materials due to their amphiphilic structure which allows them to self-assemble into bilayer or micelle structures when coming into contact with aqueous solutions [21]. Phospholipids are also able to affect the fluidity and stability of the feeding liquid’s characteristics and, consequently, the diameter and size distribution of microbubbles which could change the surface and internal morphologies of the porous film structures [22].

In the present work, the aim of this study was to investigate factors such as the effects of polymeric solution concentrations, the surfactant and phospholipid used, and the feeding liquid’s flow rate in influencing the generation of microbubbles using a T-shaped microfluidic junction device technique, and the subsequent production of porous alginate-based film structures in order to reach the optimal size and polydispersity index value of the bubbles and a highly monodisperse porous film morphology for use in tissue engineering and drug delivery systems. The high-speed camera and digital microscopy observation of the microbubbles generated using the T-shaped microfluidic junction device was done to observe the bubble formation mechanism and the bubble structure produced. The porous film structures obtained were studied using SEM, FTIR, XRD, and DSC to characterize the changes in pore size and size distribution of pore structures present in the films produced.

## 2. Materials and Methods

### 2.1. Materials

Sodium alginate, polyethyleneglycol-40 stearate (PEG-40S, with a density of 1300 kg·m^−3^), and phospholipid powders were purchased from Sigma-Aldrich (Poole, UK). Nitrogen gas was procured from Asal Gas (99.5% purity, Izmir, Turkey). Distilled water was obtained from a Millipore device (Direct-Q3-UV, Merck, Kenilworth, NJ, USA).

### 2.2. Preparation of Polymer Solutions

Polymer solutions of several different concentrations were prepared, ranging from 0.5 to 2 wt % in the present work, as shown in Table 1. To prepare a homogenous solution, sodium alginate powder was initially dissolved in distilled water followed by the addition of the polyethyleneglycol-40 stearate and phospholipids under continuous stirring for 3 h.

### 2.3. Characterization of Precursor Materials and Polymer Solutions

The precursor material characterization was done through FTIR, XRD, SEM, DSC and TGA. The details for all these processes are described in Microstructural Characterization. The surface tension and contact angle measurements were performed using a One Attension Theta Lite (Biolin Scientific, Gothenburg, Sweden). The solution viscosity was determined by Oswalt’s U-tube viscometer. A 25 mL densitometer (ISOLAB Laborgeräte GmbH, Eschau, Germany) was used to measure the density of each of the alginate, alginate–PEG-40S, and alginate–PEG-40S–phospholipid solutions at ambient temperature. To ensure the accuracy of the measurements, the average values were reported. All the measurements were conducted at ambient temperature.

### 2.4. Preparation of Alginate-Based Polymeric Microbubbles

**Figure 1:**
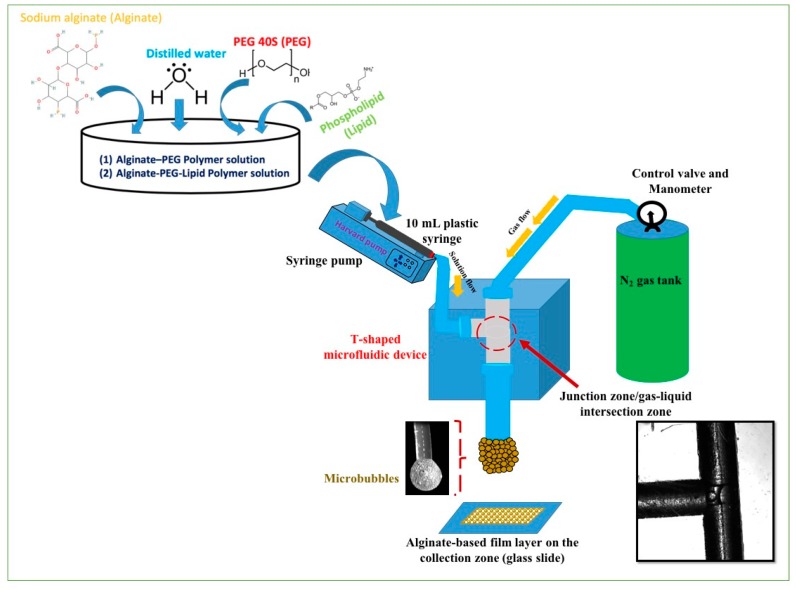
Schematic representation showing the T-shaped microfluidic junction processing of an alginate-based porous film formation from bubble bursting used in this experimental work.

The setup used in this work to achieve monodisperse microbubbles using a T-shaped microfluidic junction device is illustrated in Figure 1. Polymethyl methacrylate (PMMA) was used to produce the microfluidic platform by CNC machining. The T-shaped microfluidic junction device used in the present work consisted of inlet (15 cm) and outlet (5 cm) Teflon capillaries having an inner diameter (ID) of 200 µm. The nitrogen (N_2_) gas pressure to the vertical capillary from the gas cylinder was adjusted through a digital manometer according to the requirements to produce monodisperse bubbles. The flow of polymeric solution to the horizontal capillary was fed and controlled using a 10 mL disposable plastic syringe (BD) connected to a syringe pump (Harvard Pump 11, Elite, Harvard, Holliston, MA, USA).

Monodisperse bubble formation was achieved by inserting the polymeric solution at constant rate and gradual increase in the gas pressure so that it overcame the surface tension of the solution. The mix of both gas and polymer solution took place at the junction of the two inlet capillaries and microbubbles were produced at the gas–liquid interface. The monodispersed microbubbles produced by using a T-shaped microfluidic junction device and adjusting the polymer solution flow rate and gas pressure are shown in Figure 1. The resultant structures generated by the bubble bursting of microbubbles produced using a constant volume (10 mL) of polymer solution were collected on the glass slide at the outlet capillary and observed under an optical microscope.

### 2.5. Microstructural Characterization

The microbubbles produced were investigated through an optical microscope (Nikon, Eclipse LV 150N, Tokyo, Japan) fitted with a Clemex camera and a high-speed camera (Photron Fastcam SA8, San Diego, CA, USA). A JEOL (JSM-6390, Akishima, Japan) field emission scanning electron microscope (FE-SEM, JEOL Ltd., Akishima, Japan) was used to examine the porous film structures produced by microbubble bursting. Sputter-coating of samples were done (Edwards Sputter Coater S150B, Burgess Hill, UK) with gold for 2 min before observing through a scanning electron microscope with an accelerating voltage of 5 kV. The size variation of the microbubbles, the pore size of the porous films formed and the cross-sectional view of the porous films obtained were determined via ImageJ software (ImageJ 1.519, National Institutes of Health and the Laboratory for Optical and Computational Instrumentation (LOCI, University of Wisconsin), Madison, WI, USA).

The FTIR spectroscopy of pristine starting polymers and the prepared film was performed using a Bruker optics spectrophotometer (Tensor 37, Ettlingen, Germany). For each measurement, the spectra were obtained in the wave number ranging from 400 to 3500 cm^−1^. XRD spectra of the raw materials and the prepared film were done by a Bruker D8 Advance X-ray Diffractometer (Billerica, MA, USA), and CuKα radiation with a wavelength of 1.5406 Å was used. The scanning speed was 2θ/min. The diffraction intensity curves with 2θ from 5° to 60° were obtained. A differential scanning calorimetry instrument (Discovery DSC/250, Newcastle, UK) was used to measure the glass transition temperature of the processing materials and resultant films. For the DSC experiment, a weighed amount of samples was placed in a hermetically sealed aluminum pan heated to 250 °C with an increase of 10 °C/min.

## 3. Results

### 3.1. Physical Properties of Alginate-Based Polymeric Solutions

The viscosity and surface tension of the different polymeric solutions have greater impact on micro bubbling [14]. By increasing the concentration of polymers, the viscosity also increases, as shown in Table 2. Viscosity differs with the variation in concentration of alginate solutions [23]. Controlling the viscosity of the solution is important, because solutions with higher viscosities have an impact on the bubble bursting process [15]. An increase in PEG-40S concentration results in a decrease of surface tension while keeping the polymer concentration constant (Table 2). The surface tension of the microbubbles (shell) is another key parameter to consider because bubbles with higher surface tension cause faster film drainage [24]. The surface tension is directly related to contact angle. The surfaces having a contact angle less than 90° are hydrophilic in nature. The contact angle of solutions of different concentrations was measured and is presented in Table 2. Table 2 shows that a higher amount of alginate in the polymer solutions clearly increased surface tension and contact angle values. In contrast, an increased concentration of PEG-40S in the polymer solutions resulted in a decrease in the contact angle and surface tension values obtained (Table 2). Gaudio et al. indicated that the surface tension of the alginate solution increased linearly when increasing the concentration of polymer, which provides a description in order to determine the polymer solution’s viscosity in the nozzle [25]. In addition, Sun et al. showed that greater PEG-40S proportions in the sodium alginate polymer solution caused the contact angle of the solution to decrease, which enhanced the hydrophilicity of the polymer solution [26].

### 3.2. Production of Alginate-Based Microbubbles by Using a T-Shaped Microfluidic Junction Device

**Figure 2:**
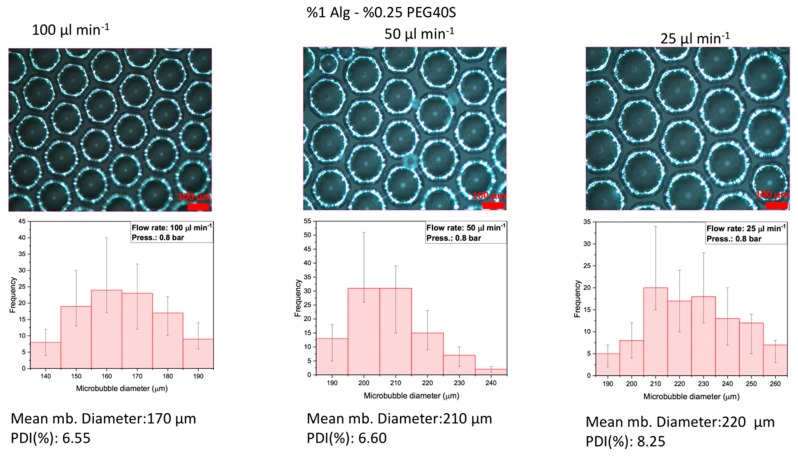
Changes of microbubble (mb) diameter and size distribution as a function of the feeding liquid’s flow rate at a constant N_2_ gas pressure of 0.8 bar. Data are presented as mean ± SD, *n* ≥ 100 microbubbles.

The solution was fed at a constant rate (100 µL·min^−1^) through a syringe pump and the pressure of N_2_ gas was adjusted up to 0.8 bar until the surface tension of the solution was overcome to produce continuous monodispersed microbubbles. The mix of both gas and polymer solution took place at the junction of the two inlet capillaries and microbubbles were produced at the liquid–gas interface, as shown in Figure 1. Monodispersed microbubbles were produced by using a T-shaped microfluidic junction device. The prime function of this device was to produce monodisperse microbubbles by using two main processing variables, namely, flow rate and gas pressure. To produce a desired microbubble size, shear forces on the solutions within the capillaries were reduced by selecting the lower flow rate, as shown in Figure 2. The physicochemical properties of the solutions, particularly the alginate solutions, may be affected by decreasing the capillary diameter or increasing the length of the microfluidic channels and increasing the flow rate, resulting in increased intensity of the shear forces to which the solutions are subjected [27].

### 3.3. Effect of Polymer Solution Flow Rate on Alginate-Based Porous Film Structures

In order to evaluate the effect of the polymer solution’s flow rate on the alginate-based porous films, the bubble bursting was investigated at different flow rates (100, 50, and 25 µL·min^−1^) with the constant polymeric concentration of sodium alginate (1%) and PEG-40S (0.25%) and gas pressure (0.8 bar). The digital microscope images captured are represented in Figure 2 and show the diameter and polydispersity index (PDI) values of the bubbles obtained. They show variations in diameter and PDI values in the microbubbles produced at different flow rates. The results revealed that the flow rate of the polymeric solution at 100 µL/min was better for producing controlled-size and highly monodispersed microbubbles. The mean diameter and PDI values of the microbubbles obtained at 100, 50, and 25 µL·min^−1^ flow rates were approximately 170, 210, and 220 µm and 6.55%, 6.63%, and 8.25%, respectively.

**Figure 3:**
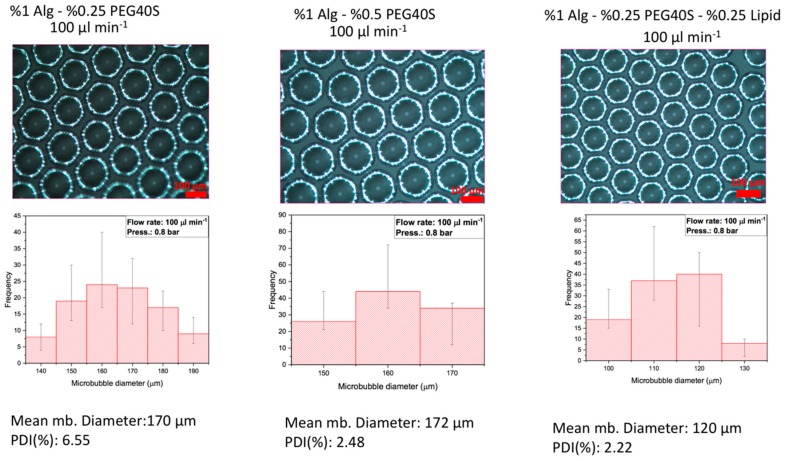
Effects of PEG-40S (surfactant) and phospholipid concentrations on the diameter and size distribution of the microbubbles obtained at a constant N_2_ gas pressure of 0.8 bar and feeding liquid’s flow rate of 100 µL·min^−1^ (phospholipid is denoted by Lipid). Data are presented as mean ± SD, *n* ≥ 100 microbubbles.

Figure 3 shows the digital microscopic images of microbubbles with variations in diameter and PDI values while keeping the flow rate constant at 100 µL·min^−1^ and varying the concentrations of alginate, PEG-40S, and phospholipid. With the concentration of %1 alginate–%0.25 PEG-40S, the mean diameter and PDI were 170 µm and 6.55%, respectively. By changing the concentration to %1 alginate–%0.5 PEG-40S, the mean diameter and PDI values changed to 172 µm and 2.48%, respectively. With the polymeric solution concentration of %1 alginate–%0.25 PEG-40S–%0.25 phospholipid, the mean diameter and PDI of the microbubbles obtained changed to 120 µm and 2.22%, respectively, showing promising results. The mean diameter and PDI ratio of the resultant 0.5 wt % PEG-40S-added and 0.25 wt % PEG-40S–phospholipid-added alginate-based microbubbles collected (Figure 3) were in the desired range and smaller than for the microbubbles seen in Figure 2.

The physical properties of various concentrations of alginate, PEG-40S, and alginate–PEG-40S solutions were assessed by measuring the contact angle, surface tension, and viscosity of the solutions. Initially, microbubbles were produced from various concentrations of alginate polymer (0.5, 1, 2, 3 wt %) and PEG-40S (0.25, 0.5, 0.75 wt %), started from the lower concentrations to check the effect of concentration of PEG-40S and viscosity of sodium alginate on porous film formation, as shown in Table 2. Depending on the results of all these physical parameters, microbubble diameter range, and polydispersity index, the concentration of alginate and PEG-40S was selected as 1 and 0.25 wt %, respectively, and 0.25 wt % phospholipid was added to the alginate–PEG-40S solution.

PEG-40S imparts stability to the microbubbles before their bursting at the outer channel of the T-shaped microfluidic device and it indicates the bubble shell thickness [28]. In the collection environment, the bubble shell thickness is minimal and then the droplets undergo shrinkage until the film drainage is completed. The bubble diameter at bursting may be affected by inhibiting or accelerating the bubble shell drainage, which would control the diameter of microbubbles that is produced within the polymeric structure after bursting [29]. The size of the microbubbles has a direct relation to the duration of stability of the microbubbles before their bursting [30].

### 3.4. Effect of the PEG-40S on Alginate-Based Porous Films

The dual role of surfactant in film formation includes controlling the gas diffusion from the bubbles and preventing the agglomeration of nearby microbubbles. The stability of microbubbles is involved in the production of films with nearly uniform morphology and porosity [31]. It is also involved in regulating the thinning process of the microbubbles leading to the production of porous polymeric films with smooth morphology [32]. Moreover, higher sodium alginate and PEG-40S concentrations produce microbubble structures with bigger dimensions between pores.

The porous structures with controlled surfaces can be obtained by regulating the ratio of polymer and surfactant. However, higher polymer concentrations can inhibit the bursting of microbubbles and result in structures lacking the proposed porous morphology and size [23]. Phospholipid addition in polymeric systems allows the incorporation of higher polymer concentrations due to their unique feature to self-assemble around the inner gaseous core of the microbubble, providing a stronger shell to the microbubbles and enabling them to incorporate into other functional units [33]. Microbubbles prepared from different concentrations of alginate–PEG-40S but with or without phospholipids involved various bursting and shrinkage mechanisms, as described in Table 2. Furthermore, microbubbles containing 0.25 wt % phospholipids showed comparatively less shrinkage in microbubble size and produced more open pores, as compared to microbubbles produced without phospholipids, as seen in Figure 2.

In this selected polymeric system, PEG-40S as an emulsifier is involved in improving the phospholipid dispersion and intercepting the agglomeration of microbubbles which in turn control the polydispersity [33]. High surfactant concentration also helps in maintaining the monodispersity of microbubbles and alignment to a significant level. The control over the surface morphology of the porous film is important due to the impact of surface properties on the cellular functions, that is, cellular adhesion, differentiation, and proliferation [34]. 

In this study, a T-shaped microfluidic junction device was used to produce well-defined porous structures by adjusting the polymeric solution concentrations. The graphical presentation of the T-shape microfluidic device-based method is shown in Figure 1. This method provides good control over the bubbles’ thinning and bursting processes. Consequently, porous structures with regular surface morphology and pore size were produced by using the T-shaped microfluidic junction device without involving any post-processing treatment. 

### 3.5. Scanning Electron Microscopy of the Resultant Film Structures

**Figure 4:**
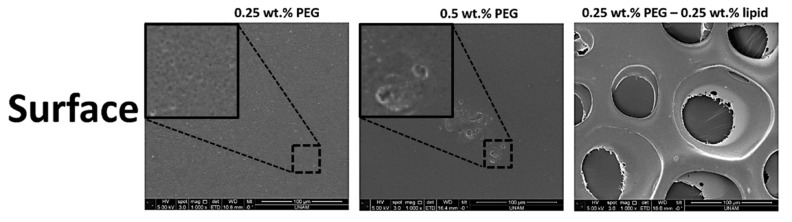
Changes of SEM morphologies of the alginate-based porous film structures formed as a function of PEG-40S (surfactant) and phospholipid concentrations at a constant N_2_ gas pressure of 0.8 bar and feeding liquid’s flow rate of 100 µL·min^−1^ (PEG-40S and phospholipid are denoted by PEG and lipid, respectively). Insets show an enlargement of the porous surface at 0.25% and 0.5% PEG samples, respectively.

**Figure 5:**
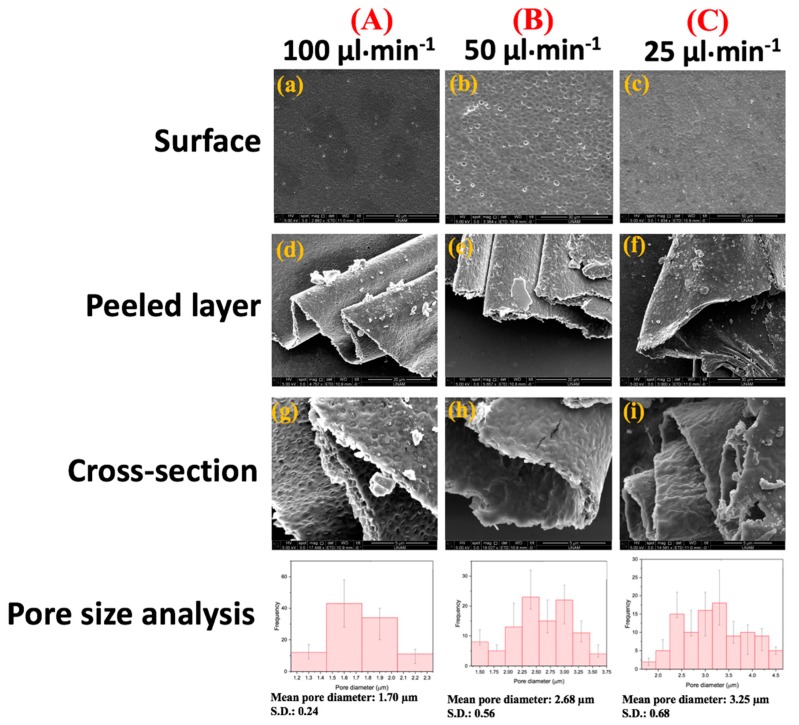
(**a–c**) SEM morphologies, (**d–f**) peeled surface, and (**g–i**) cross-sectional SEM images of the alginate-based porous films formed at a constant N_2_ gas pressure of 0.8 bar in a range of various feeding liquid’s flow rates: (**A**) 100 µL·min^−1^, (**B**) 50 µL·min^−1^, and (**C**) 25 µL·min^−1^ (the polymeric solution consisted of 1 wt % alginate and 0.25 wt % PEG-40S in this experimental work). The data in the Pore size analysis graphs are presented as the mean values of standard deviation (SD) of 100 pores.

SEM representative micrographs of surface and cross-section morphology of the produced alginate-based porous polymeric films are shown in Figure 4 and Figure 5. The variations in morphology shown in the images indicate that concentration of polymers, flow rate, and gas pressure are important factors in the preparation of optimized alginate-based porous polymeric films. Figure 4 shows the surface morphology of porous films with various concentrations of PEG-40S and the addition of phospholipid with PEG-40S. The insets in Figure 4 show remarkable size differences in pore size between the samples obtained at 0.25 and 0.5 wt % PEG and the 0.25 wt % phospholipid solutions. Films having phospholipid with PEG-40S show better bubble production with a controlled polydispersity index. We produced alginate-based porous films with variations in thickness and pore size via a drying process of the multilayers of bubbles collected (Figure 5). Figure 5 shows the surface morphology, peeled layer view, and cross-sectional view and pore size analysis of alginate porous films with constant gas pressure and polymer solution’s feeding rate. When decreasing the feeding rate of the polymeric solutions from 100 to 25 µL·min^−1^, the porous film’s thickness decreased from 1.5 to 0.5 µm. In contrast, pore size increased between 1.70 µm ± 0.24 standard deviation (SD) and 3.25 µm ± 0.68 SD for these alginate-based porous films (Figure 5). Thus, alginate-based porous film structure with variations in thickness and pore size is more sensitive to flow rate of the polymeric solution, alleviating the need for precise thickness and pore size control. It can be seen that porous films have nearly smooth surface morphology with controlled pore sizes, which was the main theme of this work and which was achieved successfully. Moreover, the film structures were prepared using a T-shaped microfluidic junction device method after the formation of microbubbles occurred (Figure 1). The resultant bubbles were then guided down an exit channel placed at the bottom, and bubble clusters were collected at the channel exit. Upon impact with the dry substrate surface, the bubble was disrupted and released the N_2_ gas while the alginate-based polymeric material formed porous film structures in the course of water evaporation (Figure 1). This combination of film formation mechanisms generated porous film structures with different pore densities and polydispersity of the pore diameter (Figure 5).

### 3.6. FTIR Results of the Precursors Used and the Alginate-Based Porous Film Structures

**Figure 6:**
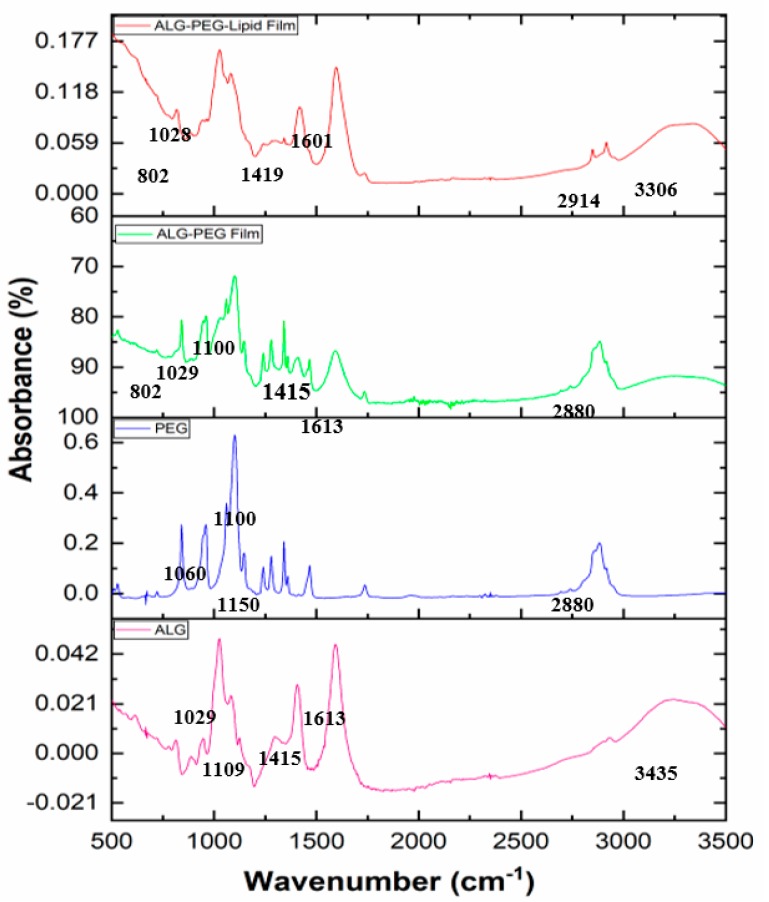
FTIR spectra of the pristine sodium alginate and PEG-40S used, and the resultant alginate–PEG and alginate–PEG–phospholipid porous films obtained (alginate, PEG-40S, alginate–PEG-40S, and alginate–PEG-40S and phospholipid are denoted by ALG, PEG, ALG–PEG, and ALG–PEG–Lipid, respectively).

In order to assess the molecular interaction of porous films, changes were monitored in the FTIR spectra of their component parts. Fourier-transform infrared spectra of the precursors, sodium alginate and PEG-40S, and of the resultant films of alginate–PEG-40S and alginate–PEG-40S–phospholipid were obtained, as shown in Figure 6. FTIR spectra of alginate showed major peaks and a strong band at 3435.09 cm^−1^ (C–H stretching), a medium sharp carboxyl salt peak at 1613 and 1415 cm^−1^ (asymmetric and symmetric COO– stretching, respectively), and then peaks at 1109.22 and 1029.25 cm^−1^ (C–O stretching). FTIR of PEG-40S showed a broader peak at 3430 cm^−1^ corresponding to the asymmetric stretching vibration of the functional group of O–H and a sharp peak at 2880 cm^−1^ (–CH_2_ stretching). At the same time, the triplet peaks of the C–O–C groups at 1150, 1110, and 1060 cm^−1^ were observed. The characteristic absorption peak at 958 cm^−1^ represents the groups of C–H. These results show similarity with the spectra already given in the literature [35,36,37]. Alginate–PEG-40S films or alginate–PEG-40S–phospholipid films show almost the same absorption peaks as pure alginate and PEG-40S, accompanied by some slight shifts. No other obvious new peaks were observed, which confirmed that there were no physical interactions and new bonds formed or strong chemical interactions occurring within the blend and porous films due to the use of the T-shaped microfluidic junction device. 

### 3.7. Differential Scanning Calorimetry Results of the Precursors Used and the Alginate-Based Porous Film Structures

**Figure 7:**
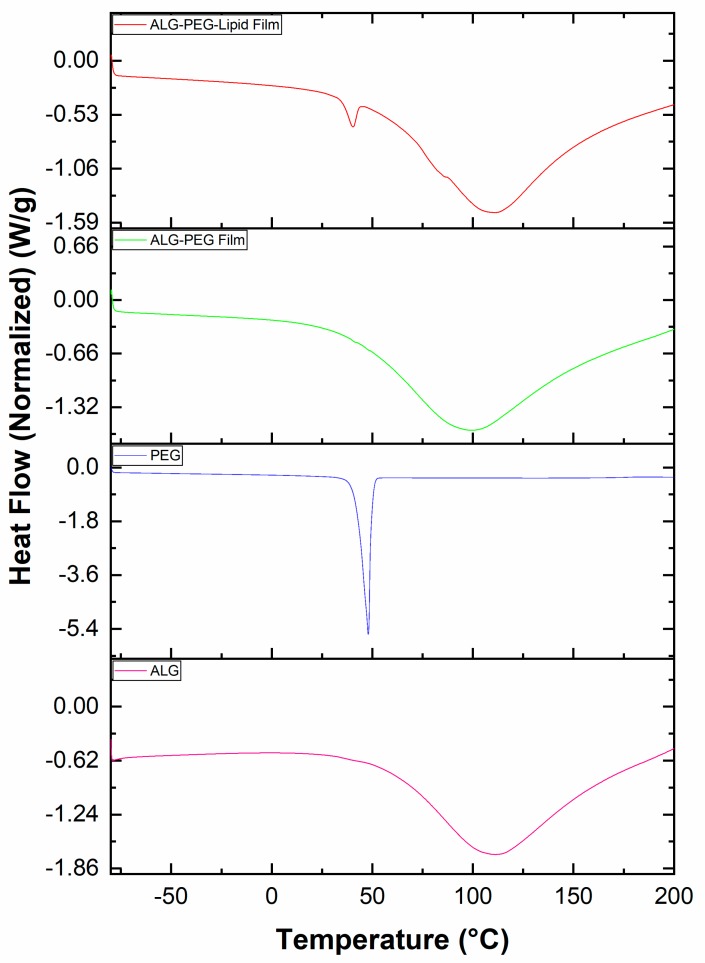
DSC spectra of the pristine sodium alginate and PEG-40S used, and the resultant alginate– PEG-40S and alginate–PEG-40S–phospholipid porous films obtained (alginate, PEG-40S, alginate–PEG-40S, and alginate–PEG-40S and phospholipid are denoted by ALG, PEG, ALG–PEG, and ALG–PEG–Lipid, respectively).

Sodium alginate exhibits an endothermic peak at 110 °C correlated with the loss of water associated with the hydrophilic groups of alginate described in Figure 7. The curve of pure PEG-40S had an endothermic peak at 48 °C starting from 36 °C, as shown in Figure 7. In the resultant film of alginate and PEG-40S, the peak is slightly shifted to a lower temperature due to the addition of PEG-40S and phospholipid to the sodium alginate and the peak area also decreases, as depicted in Figure 7. Simpliciano et al. showed that sodium alginate has an endothermic decay at 112 °C due to the removal of non-structural water, which provides information on the glass transition temperature (*T*_g_) of sodium alginate polymers [38,39]. *T*_g_ is a very significant indication of molecular chain flexibility that could influence film drainage during the solidification process [40,41]. Therefore, we believed that it was necessary to investigate the *T*_g_ value influenced by PEG-40S and phospholipid proportions to explore the thermal properties of the porous films.

### 3.8. XRD Spectra of the Precursors Used and the Alginate-Based Porous Film Structures

**Figure 8:**
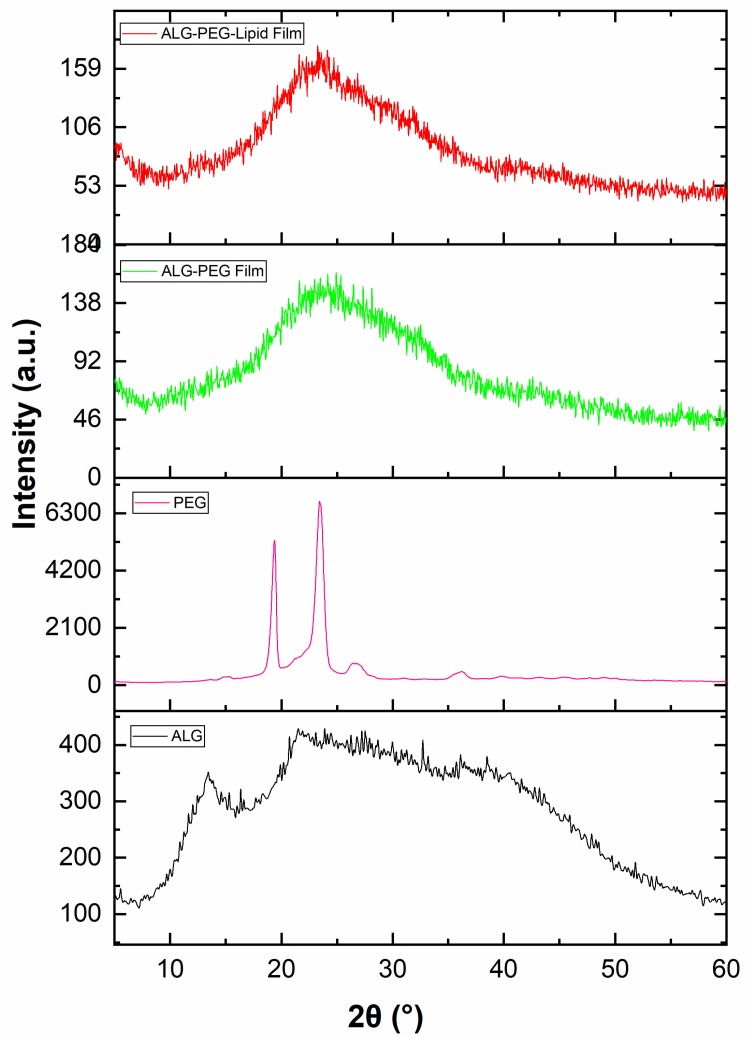
X-ray diffraction patterns of the pristine sodium alginate and PEG-40S used, and the resultant alginate–PEG-40S and alginate–PEG-40S–phospholipid porous films obtained (alginate, PEG-40S, alginate–PEG-40S, and alginate–PEG-40S, and phospholipid are denoted by ALG, PEG, ALG–PEG, and ALG–PEG–Lipid, respectively).

The XRD analysis of the samples was performed to further assess the effect of mixing and flow conditions in the T-shaped microfluidic junction device used on the crystalline structure, as presented in Figure 8. The XRD patterns of sodium alginate over the 2θ range from 10° to 60° showed that sodium alginate has a crystalline structure with peaks at 7.67°, 13.48°, 22.59°, 28.96°, and 36.64°, as shown in Figure 8. The XRD diffraction patterns of pure PEG-40S are also depicted in Figure 8, having peaks at approximately 19°, 23°, 26°, and 37° in the wave pattern belonging to the PEG-40S crystal. The wide peak between 20° and 30° shows the diffraction patterns of alginate–PEG-40S and alginate–PEG-40S–phospholipid resultant films. The same peaks in alginate–PEG-40S film and alginate–PEG-40S–phospholipid films indicate that there is no destruction in the sodium alginate and PEG-40S crystal structure and that no chemical reaction occurs between the reaction materials, resulting in no new peaks. Compared to PEG-40S, the peaks at 19° and 23° disappear in both the sodium alginate–PEG-40S film and the sodium alginate–PEG-40S–phospholipid film, respectively. Moreover, the intensity of the diffraction peak presented lower values when combining PEG-40S and phospholipid into the sodium alginate matrix. Studies conducted by researchers have reported strong interactions between sodium alginate and the reinforcement polymer material, especially complexation caused by Ca^2+^ crosslinking [36,41,42,43]. Thus, the XRD patterns provide evidence of the impregnation of PEG-40S and phospholipid into the sodium alginate matrix.

## 4. Conclusions

Highly monodispersed alginate-based microbubbles were successfully generated using a T-shaped microfluidic junction device in order to construct porous films. First, the porous films were prepared by regulating and varying a number of key parameters by polymeric solution concentration and feeding liquid’s flow rate. Alginate-based polymeric solutions with a handle of inert N_2_ gas successfully generated highly monodispersed microbubbles and, subsequently, porous film structures with varied surface morphologies (in particular, pore size and shape) were produced. Control of the microbubble thinning process was achieved by using surfactants and phospholipids, which generated alginate-based porous film structures with spherical shapes, by promoting an efficient bubble bursting process. Alginate-based polymeric films with a porous surface were grown on glass substrates. We compared the surface and internal structural morphologies of the alginate films as a function of alginate, PEG-40S, and phospholipid concentrations of the polymeric solution and feeding liquid’s flow rate in the range of 25–100 µL·min^−1^. The advanced electron microscopy indicated that the best highly monodispersed porous surface and uniform spherical internal structure were obtained for an alginate-based porous film formed at a feeding liquid’s flow rate of 100 µL·min^−1^ and a constant N_2_ gas pressure of 0.8 bar, and the best suitable polymeric solution used consisted of 1 wt % alginate and 0.25 wt % PEG-40S, respectively. The chemical and crystal structure as well as the thermal analysis confirmed that there was no damage to the T-shaped microfluidic junction device system used on the alginate-based porous film formation. Thus, this processing and forming system may have a potential to tailor the surface and internal structure features of porous films for further desired bioengineering applications, such as scaffolding and the treatment of eye diseases.

## Figures and Tables

**Table 1:** Composition of the alginate-based polymeric solutions used in this experimental work.

Solution ID	Alginate Concentration (% *w*/*v*)	PEG-40S Concentration (% *w*/*v*)	Phospholipid Concentration (% *w*/*v*)
1	0.5	-	-
2	1	-	-
3	2	-	-
4	3	-	-
5	0.5	0.25	-
6	1	0.25	-
7	2	0.5	-
8	3	0.75	-
9	1	0.25	0.25

**Table 2:** Physical properties (e.g., contact angle, surface tension, and viscosity) of the alginate-based polymeric solutions used in order to construct porous film structures in this experimental work.

Solution ID	Alginate (% *w*/*v*)	PEG-40S (% *w*/*v*)	Phospholipid (% *w*/*v*)	Contact Angle (θ)	Surface Tension (mN/m)	Viscosity (mPa·s)
1	0.5	-	-	39.68	68.69	6.51
2	1	-	-	48.03	70.09	16.94
3	2	-	-	51.48	72.1	70.51
4	3	-	-	42.91	62.1	159.4
5	0.5	0.25	-	38.08	48.3	6.86
6	1	0.25	-	46.27	58.99	16.85
7	2	0.5	-	49.84	56.73	60.6
8	3	0.75	-	34.23	57.68	155.72
9	1	0.25	0.25	46.95	49.6	16.00

## References

[1] Yuk S.H., Cho S.H., Lee H.B. (1995). pH-sensitive drug delivery system using OW emulsion. J. Control. Release.

[2] Lin S.Y., Ayres J.W. (1992). Calcium alginate beads as core carriers of 5-aminosalicylic acid. Pharm. Res..

[3] Mjahed H., Porcel C., Senger B., Chassepot A., Netter P., Gillet P., Decher G., Voegel J.-C., Schaaf P., Benkirane-Jessel N. (2008). Micro-stratified architectures based on successive stacking of alginate gel layers and poly (L-lysine)–hyaluronic acid multilayer films aimed at tissue engineering. Soft Matter.

[4] Tan W.H., Takeuchi S. (2007). Monodisperse alginate hydrogel microbeads for cell encapsulation. Adv. Mater..

[5] McMillan J.R., Akiyama M., Tanaka M., Yamamoto S., Goto M., Abe R., Sawamura D., Shimomura M., Shimizu H. (2007). Small-diameter porous Poly (ϵ-Caprolactone) films enhance adhesion and growth of human cultured epidermal keratinocyte and dermal fibroblast cells. Tissue Eng..

[6] Lawrence B.J., Madihally S.V. (2008). Cell colonization in degradable 3D porous matrices. Cell Adhes. Migr..

[7] Malafaya P.B., Silva G.A., Reis R.L. (2007). Natural–origin polymers as carriers and scaffolds for biomolecules and cell delivery in tissue engineering applications. Adv. Drug Deliv. Rev..

[8] Yao Z.-C., Yuan Q., Ahmad Z., Huang J., Li J.-S., Chang M.-W. (2017). Controlled morphing of microbubbles to beaded nanofibers via electrically forced thin film stretching. Polymers.

[9] Mehta P., Al-Kinani A.A., Arshad M.S., Singh N., van der Merwe S.M., Chang M.-W., Alany R.G., Ahmad Z. (2019). Engineering and development of chitosan-based Nanocoatings for Ocular Contact Lenses. J. Pharm. Sci..

[10] Gao G., Cui X. (2016). Three-dimensional bioprinting in tissue engineering and regenerative medicine. Biotechnol. Lett..

[11] Yao Z.-C., Wang J.-C., Wang B., Ahmad Z., Li J.-S., Chang M.-W. (2019). A novel approach for tailored medicines: Direct writing of Janus fibers. J. Drug Deliv. Sci. Technol..

[12] Wang B., Prinsen P., Wang H., Bai Z., Wang H., Luque R., Xuan J. (2017). Macroporous materials: Microfluidic fabrication, functionalization and applications. Chem. Soc. Rev..

[13] Riche C.T., Zhang C., Gupta M., Malmstadt N. (2014). Fluoropolymer surface coatings to control droplets in microfluidic devices. Lab Chip.

[14] Parhizkar M., Edirisinghe M., Stride E. (2013). Effect of operating conditions and liquid physical properties on the size of monodisperse microbubbles produced in a capillary embedded T-junction device. Microfluid. Nanofluid..

[15] Elsayed M., Huang J., Edirisinghe M. (2015). Bioinspired preparation of alginate nanoparticles using microbubble bursting. Mater. Sci. Eng. C.

[16] Hauck N., Seixas N., Centeno S., Schlüßler R., Cojoc G., Müller P., Guck J., Wöll D., Wessjohann L., Thiele J. (2018). Droplet-assisted microfluidic fabrication and characterization of multifunctional polysaccharide microgels formed by multicomponent reactions. Polymers.

[17] Jameela S., Misra A., Jayakrishnan A. (1995). Cross-linked chitosan microspheres as carriers for prolonged delivery of macromolecular drugs. J. Biomater. Sci. Polym. Ed..

[18] Moe S., Skjåk-Bræk G., Smidsrød O., Ichijo H. (1994). Calcium alginate gel fibers: Influence of alginate source and gel structure on fiber strength. J. Appl. Polym. Sci..

[19] King A. (1983). Brown seaweed extracts (alginates). Food Hydrocoll..

[20] Swamy T.M., Ramaraj B. (2010). Siddaramaiah, sodium alginate and poly (ethylene glycol) blends: Thermal and morphological behaviors. J. Macromol. Sci. Part A Pure Appl. Chem..

[21] Schuster B., Pum D., Braha O., Bayley H., Sleytr U.B. (1998). Self-assembled α-hemolysin pores in an S-layer-supported lipid bilayer. Biochim. Biophys. Acta (BBA) Biomembr..

[22] Zhao Y.-Z., Luo Y.-K., Lu C.-T., Xu J.-F., Tang J., Zhang M., Zhang Y., Liang H.-D. (2008). Phospholipids-based microbubbles sonoporation pore size and reseal of cell membrane cultured in vitro. J. Drug Target..

[23] Amoyav B., Benny O. (2019). Microfluidic based fabrication and characterization of highly porous polymeric microspheres. Polymers.

[24] Kočárková H., Rouyer F., Pigeonneau F. (2013). Film drainage of viscous liquid on top of bare bubble: Influence of the Bond number. Phys. Fluids.

[25] Del Gaudio P., Colombo P., Colombo G., Russo P., Sonvico F. (2005). Mechanisms of form and disintegration of alginate beads obtained by prilling. Int. J. Pharm..

[26] Sun F., Guo J., Liu Y., Yu Y. (2019). Preparation, characterizations and properties of sodium alginate grafted acrylonitrile/polyethylene glycol electrospun nanofibers. Int. J. Biol. Macromol..

[27] Hallow D.M., Seeger R.A., Kamaev P.P., Prado G.R., LaPlaca M.C., Prausnitz M.R. (2008). Shear-induced intracellular loading of cells with molecules by controlled microfluidics. Biotechnol. Bioeng..

[28] Lhuissier H., Villermaux E. (2012). Bursting bubble aerosols. J. Fluid Mech..

[29] Li D. (1996). Coalescence between small bubbles: Effects of surface tension gradient and surface viscosities. J. Colloid Interface Sci..

[30] Filho W.D.A., Schneider F.K., Morales R.E. (2012). Evaluation of stability and size distribution of sunflower oil-coated micro bubbles for localized drug delivery. Biomed. Eng. Online.

[31] Huerre A., Miralles V., Jullien M.-C. (2014). Bubbles and foams in microfluidics. Soft Matter.

[32] Hahn P.S., Chen J.D., Slattery J. (1985). Effects of London-van der Waals forces on the thinning and rupture of a dimpled liquid film as a small drop or bubble approaches a fluid-fluid interface. AIChE J..

[33] Borden M.A., Longo M.L. (2002). Dissolution behavior of lipid monolayer-coated, air-filled microbubbles: Effect of lipid hydrophobic chain length. Langmuir.

[34] Jang Y., Park S., Char K. (2011). Functionalization of polymer multilayer thin films for novel biomedical applications. Korean J. Chem. Eng..

[35] Qi Y., Jiang M., Cui Y.-L., Zhao L., Zhou X. (2015). Synthesis of quercetin loaded nanoparticles based on alginate for Pb (II) adsorption in aqueous solution. Nanoscale Res. Lett..

[36] Gao J., Tao W., Chen D., Guo X., Chen Y., Jiang Y. (2018). High performance shape-stabilized phase change material with nanoflower-like wrinkled mesoporous silica encapsulating polyethylene glycol: Preparation and thermal properties. Nanomaterials.

[37] Shen Z., Wang J., Lu D., Li Q., Zhou C., Zhu Y., Hu X. (2015). Synthesis and properties of flexible polyurethane using ferric catalyst for hypopharyngeal tissue engineering. BioMed Res. Int..

[38] Simpliciano C., Clark L., Asi B., Chu N., Mercado M., Diaz S., Goedert M., Mobed-Miremadi M. (2013). Cross-linked alginate film pore size determination using atomic force microscopy and validation using diffusivity determinations. J. Surf. Eng. Mater. Adv. Technol..

[39] Dudek G., Turczyn R. (2018). New type of alginate/chitosan microparticle membranes for highly efficient pervaporative dehydration of ethanol. RSC Adv..

[40] Guo Y., Ma J., Lv Z., Zhao N., Wang L., Li Q. (2018). The effect of plasticizer on the shape memory properties of poly (lactide acid)/poly (ethylene glycol) blends. J. Mater. Res..

[41] Chen Y., Zhu Y., Wang J., Lv M., Zhang X., Gao J., Zhang Z., Lei H. (2017). Novel shape-stabilized phase change materials composed of polyethylene glycol/nonsurfactant-templated mesoporous silica: Preparation and thermal properties. JOM.

[42] Aprilliza M. (2017). Characterization and Properties of Sodium Alginate from Brown Algae Used as an Ecofriendly Superabsorbent.

[43] Qu B., Li J.-R., Xiao H.-N., He B.-H., Qian L.-Y. (2016). Facile preparation and characterization of sodium alginate/graphite conductive composite hydrogel. Polym. Compos..

